# Prognostic Nomogram of Prognosis-Related Genes and Clinicopathological Characteristics to Predict the 5-Year Survival Rate of Colon Cancer Patients

**DOI:** 10.3389/fsurg.2021.681721

**Published:** 2021-06-16

**Authors:** Chao Huang, Jiefeng Zhao, Zhengming Zhu

**Affiliations:** Department of Gastrointestinal Surgery, The Second Affiliated Hospital of Nanchang University, Nanchang, China

**Keywords:** colon cancer, prognostic genes, clinicopathological characteristics, overall survival, nomogram

## Abstract

**Background:** The Cancer Genome Atlas (TCGA) has established a genome-wide gene expression profile, increasing our understanding of the impact of tumor heredity on clinical outcomes. The aim of this study was to construct a nomogram using data from the TCGA regarding prognosis-related genes and clinicopathological characteristics to predict the 5-years survival rate of colon cancer (CC) patients.

**Methods:** Kaplan–Meier and Cox regression analyses were used to identify genes associated with the 5-years survival rate of CC patients. Cox regression was used to analyze the relationship between the clinicopathological features and prognostic genes and overall survival rates in patients with CC and to identify independent risk factors for the prognosis of CC patients. A nomogram for predicting the 5-years survival rate of CC patients was constructed by R software.

**Results:** A total of eight genes (KCNJ14, CILP2, ATP6V1G2, GABRD, RIMKLB, SIX2, PLEKHA8P1, and MPP2) related to the 5-years survival of rate CC patients were identified. Age, stage, and PLEKHA8P1 were independent risk factors for the 5-years survival rate in patients with CC. The accuracy, sensitivity and specificity of the nomogram model constructed by age, TNM staging, and PLEKHA8P1 for predicting the 5-years survival of rate CC patients were 83.3, 83.97, and 85.79%, respectively.

**Conclusion:** The nomogram can correctly predict the 5-year survival rate of patients with CC, thus aiding the individualized decision-making process for patients with CC.

## Introduction

Colon cancer (CC) ranks third in incidence and second in mortality rates (1). Surgical treatment is the main method for managing CC to prolong survival time (2, 3). Adjuvant chemoradiotherapy can also significantly improve the prognosis of CC (4, 5). The 5-years survival rates of patients with stage I, II, and III CC are ~93, 80, and 60%, respectively (6). The American Joint Committee on Cancer (AJCC) TNM staging system is widely used to assess the prognosis of patients with CC (5). However, the prognosis of patients with CC at the same stage varies widely, and the accuracy of TMN staging as a predictive approach has certain limitations (7, 8). Therefore, another approach is needed to identify patients with poor prognosis to allow for the development of individualized treatment and monitoring approaches. Nomograms can provide an overall probability of a specific outcome for an individual patient and provide more accurate predictions than traditional staging systems, thereby improving personalized treatment decisions (9, 10). Previously developed microarray techniques can be used to predict the prognosis of many types of cancer (11–13). Previous studies have shown that gene expression profiles have certain application prospects in predicting patients' long-term prognosis (14). Meanwhile, prognostic gene expression profiles of colorectal cancer patients from tumor samples and adjacent normal mucosa have been described (15–17). Relevant studies have indicated that gene expression characteristics can improve the accuracy of prognosis prediction for stage II and III colorectal cancer (18, 19). However, few studies have combined prognostic genes with clinicopathological features to predict the long-term survival of patients with CC. In addition, The Cancer Genome Atlas (TCGA) has established a genome-wide gene expression profile, increasing our understanding of the impact of tumor heredity on clinical outcomes (20). Therefore, the aim of this study was to construct a nomogram using data from the TCGA regarding prognosis-related genes and clinicopathological characteristics to predict the 5-years survival rate of CC patients, thus providing an important basis for individualized decision-making for patients with CC.

## Materials and Methods

### Data Download and Processing

RNA sequencing results from 437 tissues and 382 human colon adenoma and adenocarcinoma samples were obtained from the TCGA database (https://portal.gdc.cancer.gov). RNA sequencing results from 39 normal samples and 398 cancer samples were combined into a single matrix file using scripts in the Perl language (http://www.perl.org/). The Ensembl database (http://www.ensembl.org/index.html) was then used to convert the Ensembl ID in the matrix file to the gene name. Moreover, the clinical data of 385 cases were downloaded, and relevant clinical data were extracted.

### Identification of Prognostic-Related Genes

First, Kaplan–Meier and Cox regression analyses were used to screen for genes associated with the 5-years survival of rate CC patients, and a *P* < 0.05 was used to define statistical significance. Next, the survivalROC package in R language was used to identify genes that were associated with 5-years survival and had an area under the curve (AUC) >0.6.

### Survival Analysis

To determine the relationship between prognostic genes and CC survival, we used the survival package in R language for the survival analysis of the prognostic genes. The relationship between the clinicopathological characteristics and prognosis-related genes and the overall survival of patients with CC was analyzed by a univariate analysis. The factors affecting CC survival in the univariate analysis were analyzed by multivariate Cox regression to identify independent risk factors for CC prognosis.

### ROC Curve Analysis

To determine the accuracy of the combined factors to predict the 5-years survival rate of CC patients and the cutoff value of prognostic genes, we used the survivalROC package in R language for analysis. In addition, the sensitivity and specificity of the combined factors were calculated using the survivalROC package in R language.

### Construction of Nomogram

The combined factors that predict the most accurate prognosis of CC were used to construct a nomogram model for predicting the 5-years survival rate of CC patients using the rms package in R language, and scores for various indicators were obtained. The scores corresponding to the indicators were added to obtain a total score; the higher the total score, the lower the 5-years survival rate of CC patients. Meanwhile, the survivalROC package was used to calculate the sensitivity and specificity of the model to evaluate its clinical value. Moreover, the concordance index (C-index) was calculated to evaluate the performance of the model prediction results, and the calibration curve was plotted to observe the relationship between the predicted probability and the actual incidence (21, 22).

## Results

### Clinical Characteristics of Patients

From the clinical data of 385 patients, the patients' age, sex, stage, TNM staging, survival time and survival status were extracted. After deleting samples with incomplete clinical data, a total of 364 cases were retained for further analysis (Table 1).

**Table 1:** Clinical characteristics of patients with colon cancer.

**Clinical characteristics**	***n* (%)**
**Age**	
≤65 years	148 (40.66)
>65 years	216 (59.34)
**Sex**	
Male	192 (52.75)
Female	172 (47.25)
**Stage**	
I	63 (17.31)
II	146 (40.11)
III	102 (28.02)
IV	53 (14.56)
**T classification**	
T1	7 (1.92)
T2	64 (17.58)
T3	251 (68.96)
T4	42 (11.54)
**M classification**	
M0	278 (76.37)
M1	53 (14.56)
Mx	33 (9.07)
**N classification**	
N0	216 (59.34)
N1	85 (23.35)
N2	63 (17.31)
**Survival status**	
Death	65 (17.86)
Survival	299 (82.14)

### Prognosis and Survival Analysis

**Figure 1:**
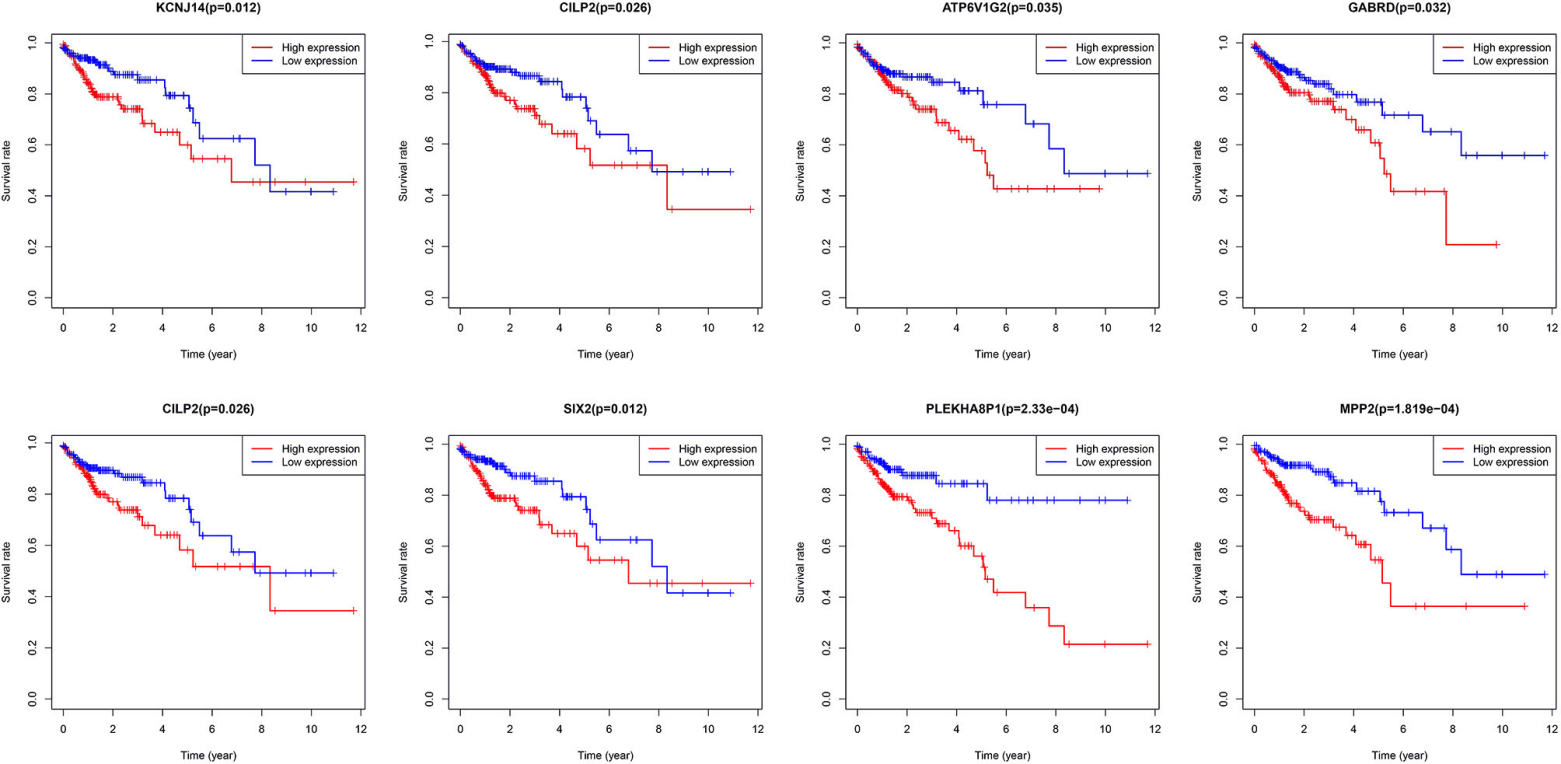
Survival curves of eight genes, KCNJ14, CILP2, ATP6V1G2, GABRD, RIMKLB, SIX2, PLEKHA8P1, and MPP2.

**Figure 2:**
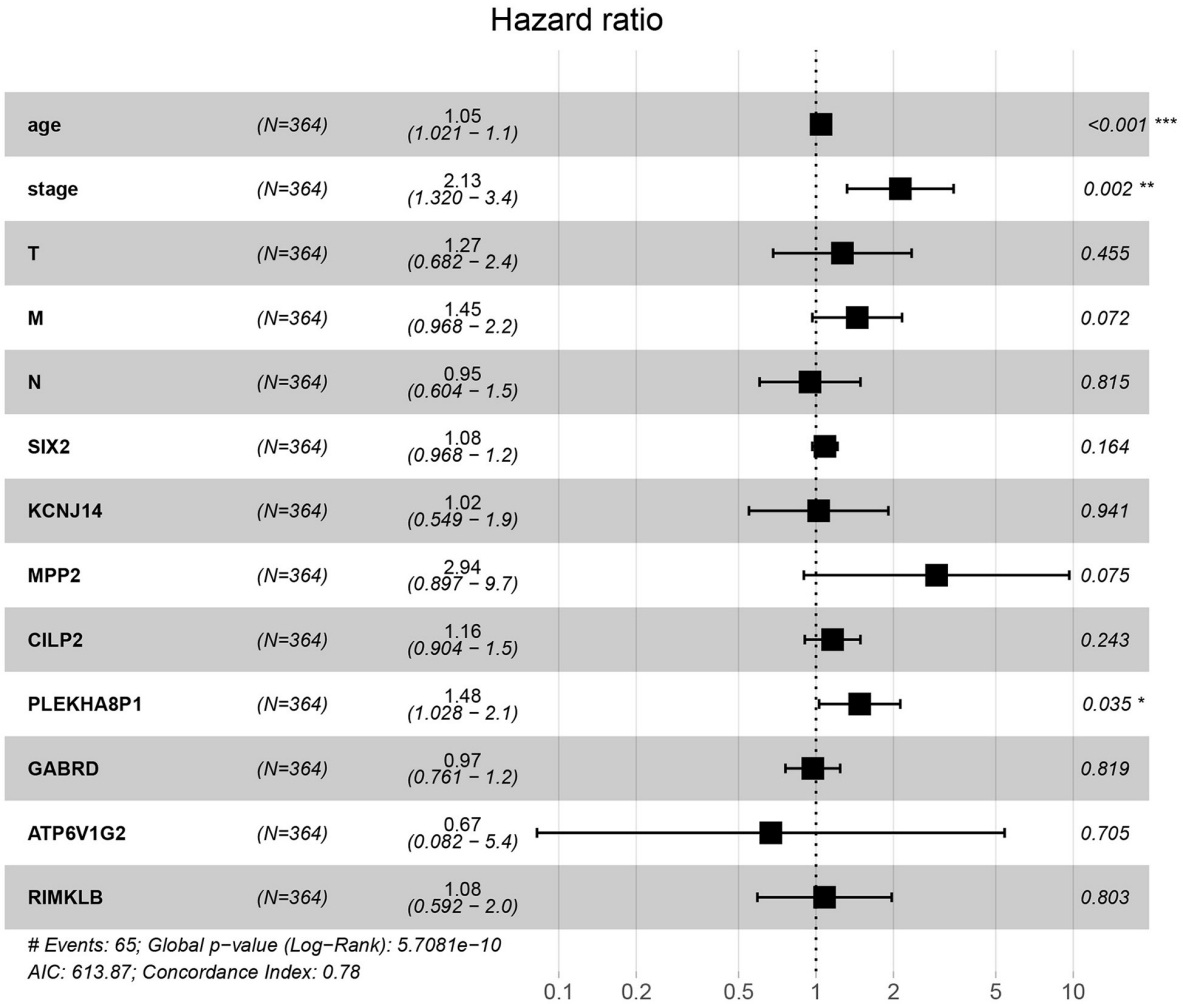
Forest plot of the correlation of prognostic-related genes and clinicopathological features with overall survival among patients with colon cancer. *P < 0.05, **P < 0.01, ***P < 0.001.

**Figure 3:**
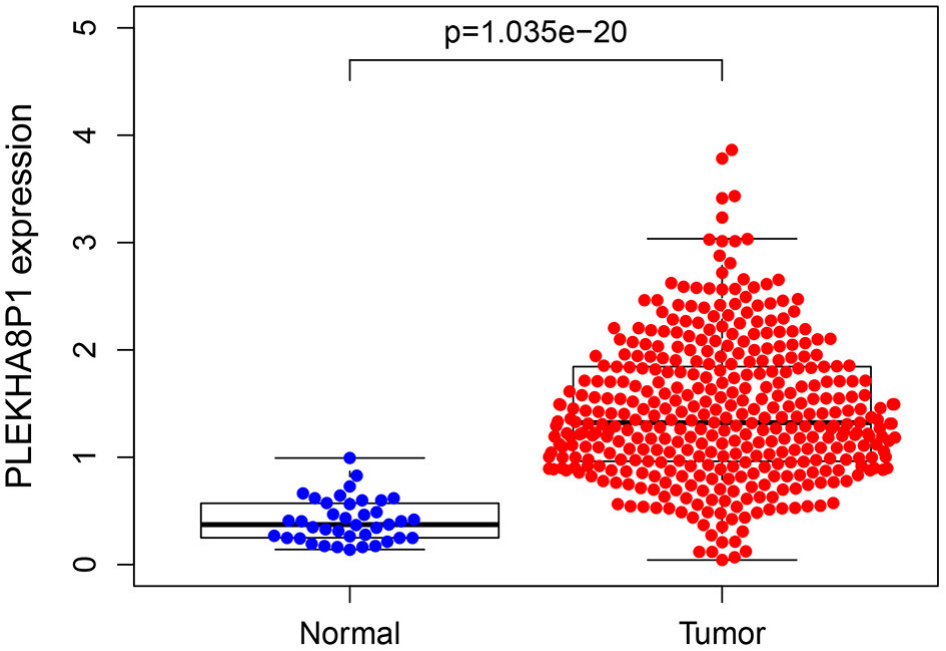
Expression level of PLEKHA8P1 in colon tumors and normal tissues.

A total of eight genes (KCNJ14, CILP2, ATP6V1G2, GABRD, RIMKLB, SIX2, PLEKHA8P1, and MPP2) associated with the 5-years survival rate of CC patients were identified (Table 2). The Kaplan–Meier analysis showed that all eight genes were correlated with the prognosis of CC (all *P* < 0.05) (Figure 1). The univariate analysis showed that the factors related to the overall survival rate of patients with CC were as follows: age (*P* = 0.010), stage (*P* < 0.001), T classification (*P* < 0.001), M classification (*P* < 0.001), N classification (*P* < 0.001), KCNJ14 (*P* < 0.001), CILP2 (*P* = 0.014), ATP6V1G2 (*P* < 0.001), GABRD (*P* = 0.005), RIMKLB (*P* = 0.008), SIX2 (*P* < 0.001), PLEKHA8P1 (*P* = 0.009), and MPP2 (*P* < 0.001). The multivariate Cox regression analysis showed that age (*P* = 0.010), stage (*P* < 0.001), and PLEKHA8P1 (*P* = 0.009) were independent risk factors for the prognosis of patients with CC (Table 3 and Figure 2). Moreover, PLEKHA8P1 was highly expressed in colon tumors (*P* < 0.001) (Figure 3).

**Table 2:** Prognostic-related gene in patients with colon cancer.

**Gene**	**Log-rank *p-*value**	**Hazard ratio (95%CI)**	**Cox *p-*value**	**AUC**
KCNJ14	0.021	2.04 (1.19–3.51)	0.009	0.611
CILP2	0.025	1.25 (1.05–1.49)	0.014	0.613
ATP6V1G2	0.020	6.18 (2.48–15.36)	0.000	0.627
GABRD	0.017	1.21 (1.06–1.38)	0.005	0.634
RIMKLB	0.008	1.65 (1.14–2.39)	0.008	0.635
SIX2	0.013	1.17 (1.09–1.26)	0.000	0.638
PLEKHA8P1	0.000	1.54 (1.12–2.12)	0.009	0.656
MPP2	0.000	4.11 (2.13–7.92)	0.000	0.710

**Table 3:** Univariate analysis and multivariate analysis of the correlation of prognostic-related genes with overall survival among patients with colon cancer.

**Clinicopathologic variable**	**Univariate analysis**		**Multivariate analysis**	
	**HR (95% CI)**	***p*-value**	**HR (95% CI)**	***p*-value**
Age	1.03 (1.01–1.05)	0.010	1.05 (1.02–1.07)	0.000
Sex	1.33 (0.81–2.18)	0.267		
Stage	2.26 (1.70–3.00)	0.000	2.13 (1.32–3.43)	0.002
T classification	2.68 (1.64–4.37)	0.000	1.27 (0.68–2.35)	0.455
M classification	1.85 (1.37–2.51)	0.000	1.45 (0.97–2.16)	0.072
N classification	1.99 (1.50–2.65)	0.000	0.95 (0.60–1.49)	0.815
KCNJ14	2.05 (1.19–3.52)	0.009	1.02 (0.55–1.91)	0.941
CILP2	1.25 (1.05–1.49)	0.012	1.16 (0.90–1.49)	0.243
ATP6V1G2	5.61 (2.20–14.30)	0.000	0.67 (0.08–5.41)	0.705
GABRD	1.21 (1.06–1.38)	0.005	0.97 (0.76–1.24)	0.819
RIMKLB	1.31 (1.01–1.71)	0.043	1.08 (0.59–1.97)	0.803
SIX2	1.17 (1.09–1.26)	0.000	1.08 (0.97–1.21)	0.164
PLEKHA8P1	1.54 (1.12–2.13)	0.008	1.48 (1.03–2.13)	0.035
MPP2	4.08 (2.12–7.83)	0.000	2.94 (0.90–9.65)	0.075

### ROC Curve Analysis

**Figure 4:**
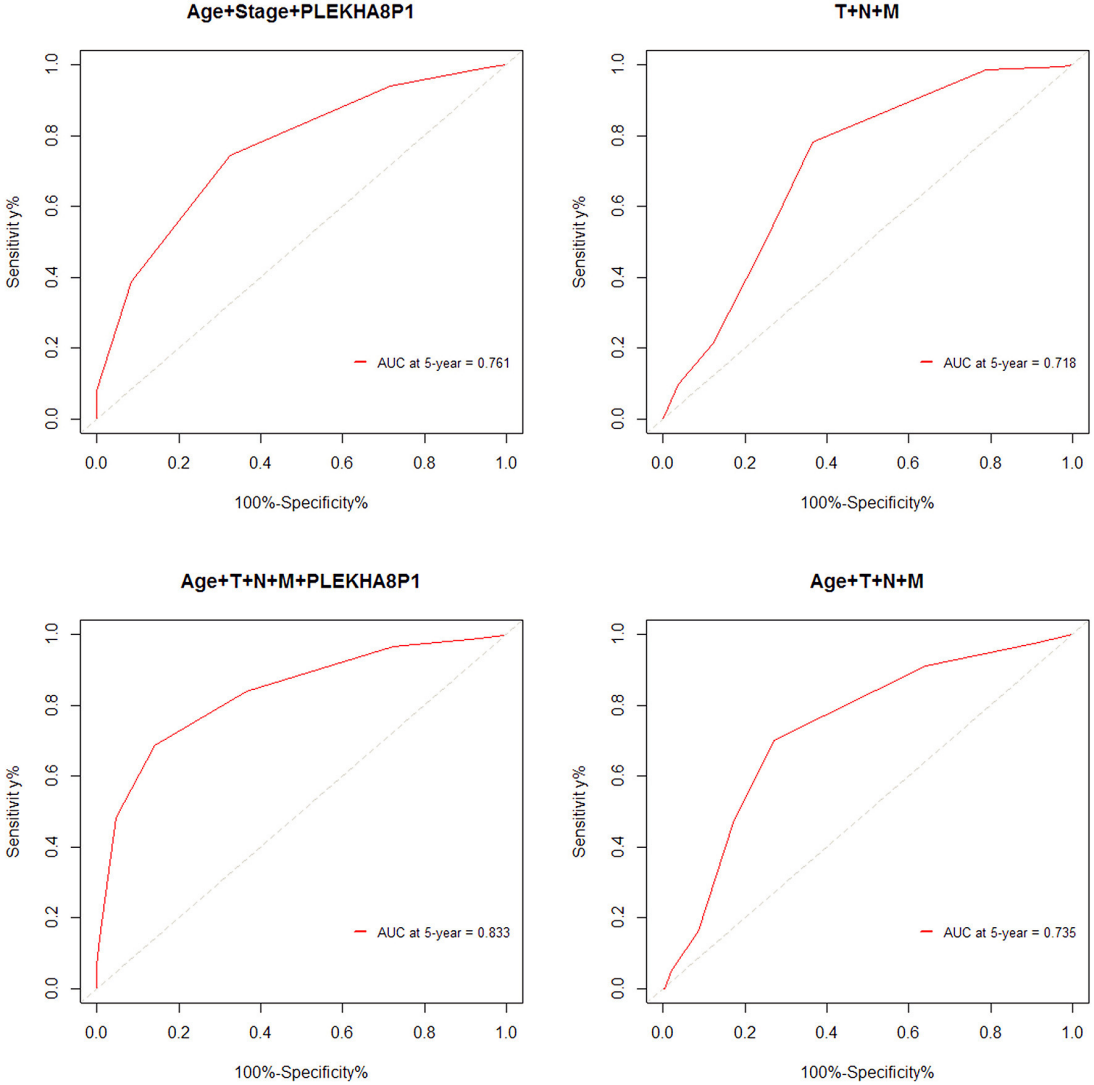
ROC curve for evaluating the 5-years survival rate of colon cancer patients based on combined factors.

The cutoff values for age and PLEKHA8P1 to evaluate 5-years survival in patients with CC were 65 years and 1.545, respectively. The AUC, sensitivity and specificity of age combined stage and PLEKHA8P1 for assessing the 5-year survival rate of CC patients were 0.761, 74.50, 67.40%, respectively. The AUC, sensitivity and specificity of TNM staging for assessing the 5-years survival rate of CC patients were 0.718, 78.25, and 63.57%, respectively. The AUC, sensitivity and specificity of age combined with TNM staging and PLEKHA8P1 for assessing the 5-years survival rate of CC patients were 0.833, 83.97, and 85.79%, respectively. The AUC, sensitivity and specificity of age combined with TNM staging for assessing the 5-years survival rate of CC patients were 0.735, 70.21, and 73.06%, respectively (Figure 4). These results indicated that age combined with TNM staging and PLEKHA8P1 were most accurate for evaluating the 5-years survival rate of CC patients.

### Construction of Nomogram

**Figure 5:**
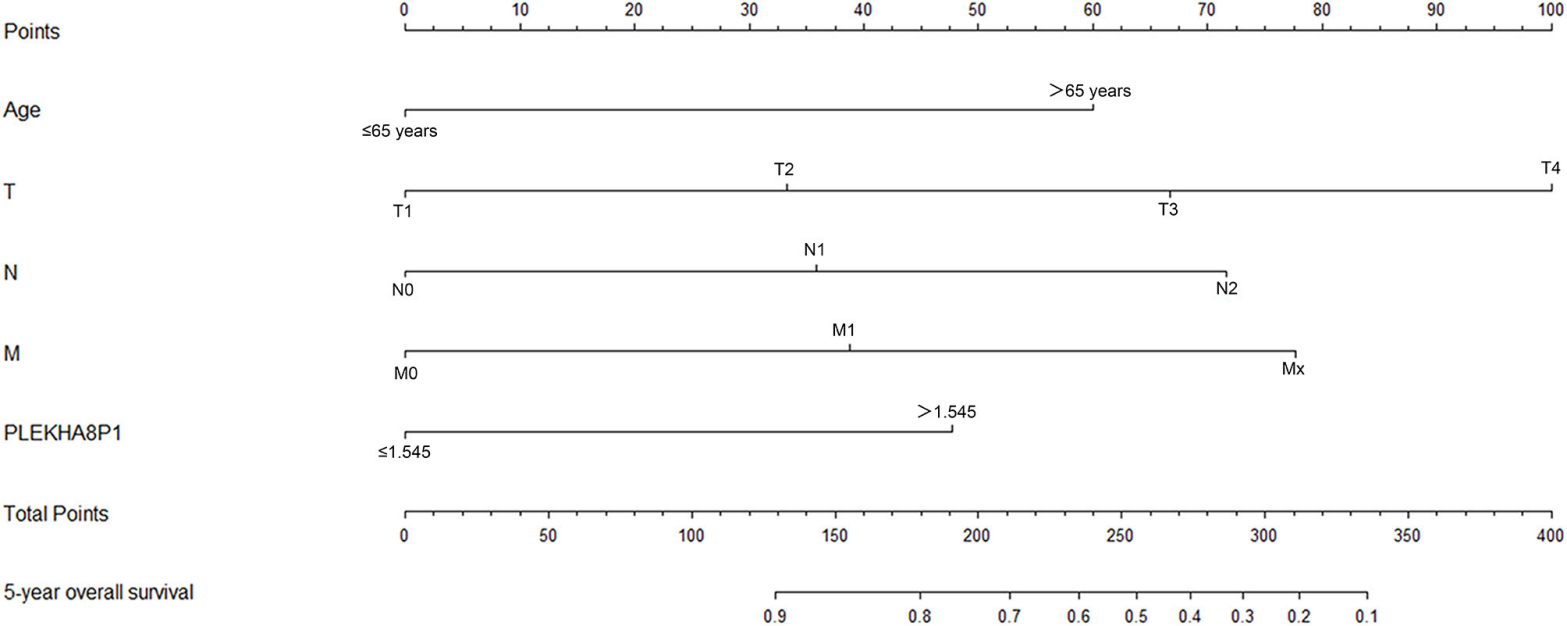
Nomogram of the logistic regression model constructed by age, TNM staging, and PLEKHA8P1.

**Figure 6:**
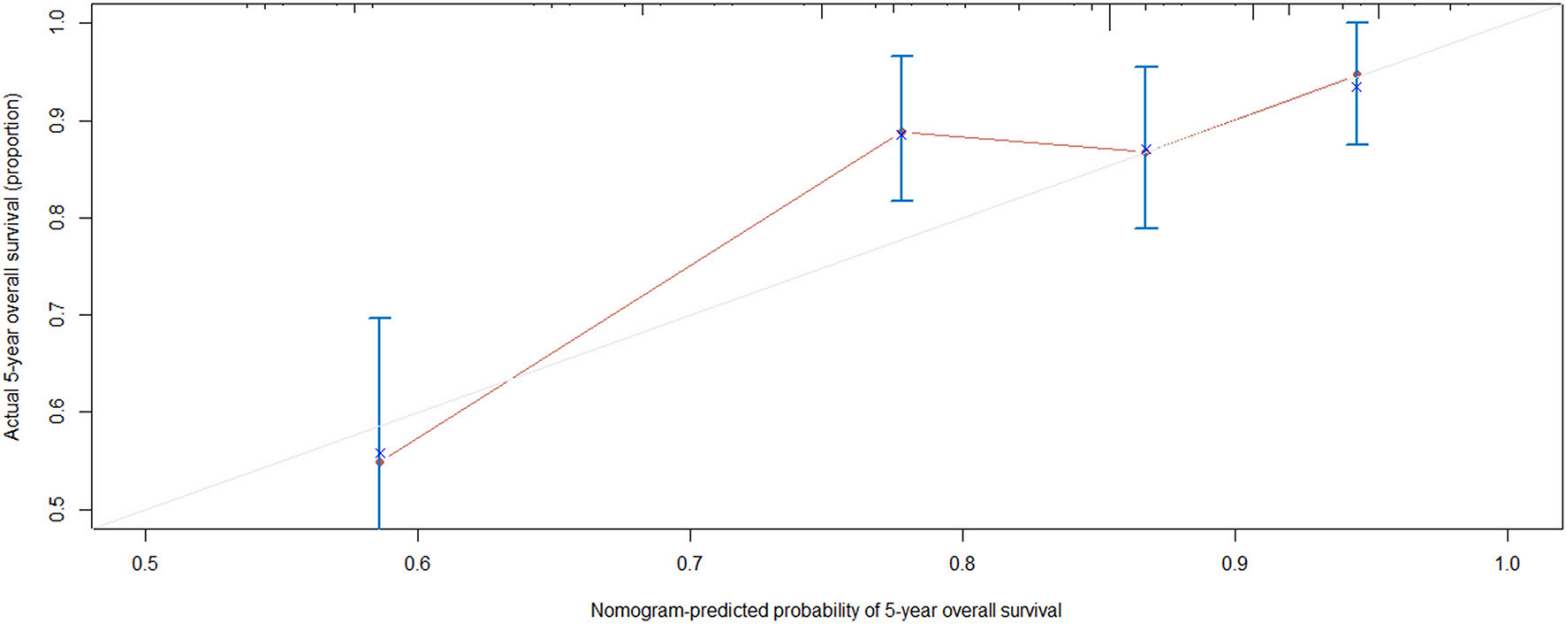
Calibration curve. Nomogram-predicted probability of 5-years overall survival is plotted on the x-axis; actual 5-years overall survival is plotted on the y-axis. Shorter distance between two curves indicates higher accuracy.

The rms package in R language was used to construct a logistic regression model constructed by age, TNM staging and PLEKHA8P1, and the C-index for evaluation was 0.74, indicating that the prediction model was accurate. Then, the plotting function was constructed, and the nomogram was plotted (Figure 5). A score of age ≤65 years was 0 points, while a score of age >65 years was 60 points; a score of T1 was 0 points; a score of T2 was 33 points; a score of T3 was 67 points; a score of T4 was 100 points; a score of N0 was 0 points; a score of N1 was 36 points; a score of N2 was 72 points; a score of M0 was 0 points; a score of M1 was 39 points; a score of Mx was 78 points; and a score of PLEKHA8P1 ≤ 1.545 U/ml was 0 points, while a score of PLEKHA8P1 > 1.545 U/ml was 48 points. The highest score was 358 points, suggesting that the 5-years survival probability of patients with CC was <10%. The probability of 5-years survival of CC can be predicted based on the total points (Table 4). The accuracy, sensitivity and specificity of this prediction model were 83.30, 83.97, and 85.79%, respectively, indicating the validity of the model. The calibration curve was closer to the ideal curve, which indicated that the prediction was in good agreement with the actual results (Figure 6).

**Table 4:** Relationship between total points and 5-years overall survival in colon cancer.

**Total points**	**5-years overall survival**
>336	<10%
312–336	10–20%
292–311	21–30%
274–291	31–40%
255–273	41–50%
235–254	51–60%
211–234	61–70%
179–210	71–80%
129–178	81–90%
<129	>90%

## Discussion

This study is the first to combine prognostic genes and clinicopathological characteristics of CC to predict the 5-years survival rate of CC patients. We first performed a series of analyses to identify genes that significantly affected 5-years survival in CC. Then, the relationship between these genes and clinicopathological characteristics and the overall survival rate of CC was analyzed, and independent risk factors for CC survival were identified. Finally, a logistic regression model was constructed based on the AUC of the combined factors, and a nomogram was drawn to predict the 5-years overall survival rate of CC patients. This study found that age, stage, and PLEKHA8P1 were independent risk factors for the 5-years survival rate in patients with CC. PLEKHA8P1 belongs to the pseudogene family. Only ~2% of the genes in the human genome encode proteins. Non-coding RNAs include microRNAs, long non-coding RNAs, and pseudogenes (23–25). Currently, the functions and mechanisms of lncRNAs and pseudogenes have not been fully elucidated (24–26). However, an increasing number of studies have shown that pseudogenes have important biological functions (27, 28). In the process of homologous recombination, pseudogenes may result in the loss of some bases, thus affecting the transcription level of genes (29). Pseudogenes can also induce endogenous small interfering RNAs to inhibit the expression of functional genes (25). Pseudogene RNAs can play a regulatory role as competing endogenous RNAs (26, 30). On the other hand, the results of an increasing number of studies have indicated that pseudogenes play a crucial role in cancer. Chen et al. (31) found that the pseudogene CTNNAP1 promotes the growth of human tumors by regulating the expression of its homologous gene, CTNNA1. Lin et al. (32) showed that the pseudogene OCT4-pg could inhibit the growth and differentiation of mesenchymal stem cells. Rutnam et al. indicated that the pseudogene TUSC2p1 protects the expression of the tumor suppressor gene TUSC2 by competitively binding with miRNA, thereby inhibiting the proliferation of breast cancer cells (33). Poliseno et al. (25) demonstrated that the pseudogene PTENP1 had the ability to produce the corresponding mRNA and can interact with the transcription products of the parent gene PTEN, thus playing a role in inhibiting cell growth. Poliseno et al. (34) also found deletion of the pseudogene PTENP1 in some CC, gastric cancer and malignant melanoma. Moreover, the expression of some pseudogenes is related to the staging and grading of cancer and can be a molecular marker for the prognosis of cancer. The increased expression level of the pseudogene OCT4-pgq1 was closely associated with poor prognosis in gastric cancer and could lead to worse overall patient survival rates (35). PLEKHA8P1 expression was significantly correlated with the monthly survival rate and monthly disease-free survival rate of renal cell carcinoma patients, suggesting that its expression changes play a key role in predicting the prognosis of renal cell carcinoma (36). This study also showed that PLEKHA8P1 was significantly associated with the 5-years survival rate of CC patients and was highly expressed in colon tumors.

The American Joint Committee on Cancer TNM staging system is widely used for the prognostic evaluation of CC patients (5). However, Liu et al. (8) indicated that the MSKCC nomogram was better than the AJCC staging system for predicting the 5-years survival rate, and the C-index of the MSKCC nomogram in the studied Chinese cohort was 0.71. Weiser et al. (37) demonstrated that a prognostic model including prognostic factors was superior to the current AJCC system, and its C-index increased from 0.60 to 0.68. The applicability of gene expression profiles for predicting the prognosis of colorectal cancer patients has been demonstrated in several studies (19, 38, 39). Barrier et al. (38) showed that microarray gene expression profile analysis can predict the prognosis of patients with stage II CC. Lee et al. (40) found that a nomogram model including TNM staging and genetic risk score obtained from the TCGA database could successfully predict the overall survival rate of colorectal cancer patients, and its C-index was higher than that of TNM staging alone (0.75 vs. 0.69). The prognostic prediction model constructed by pathologic M combined with pathologic T had a prognostic prediction efficiency with a 5-years AUC of 0.712 and C-index of 0.680 for patients with colon adenocarcinoma (41). Another prognostic model composed of six significant prognostic factors (age, first-degree relative cancer history, differentiation grade, vessel/nerve invasion, TNM stage, and HALP) had a 5-years AUC of 0.73 for patients with locally advanced colorectal cancer (42). The prognostic nomogram constructed by age, sex, histological grade, T stage, number of lymph nodes retrieved, tumor size and N stage had a 5-years AUC of 0.729 for patients with non-metastatic CC (43). In this study, the accuracy, sensitivity and specificity of age combined with TNM staging and PLEKHA8P1 for predicting the 5-years survival rate of CC patients were higher than those of the TNM staging system. In addition, the C-index of the model constructed by age, TNM staging, and PLEKHA8P1 for predicting the 5-years survival rate was 0.74, and its accuracy, sensitivity, and specificity were 83.3, 83.97, and 85.79%, respectively, indicating that the model has high validity. There are some limitations in this study. First, the mRNA gene expression value is difficult to obtain due to the high cost in clinical practice. However, when the cost is reduced, this approach could be widely used in clinical practice. Second, other prognostic factors, such as tumor markers and inflammatory markers, were not included.

In conclusion, age, PLEKHA8P1 and stage were risk factors for poor patient prognosis in CC. The nomogram model constructed by age, TNM staging, and PLEKHA8P1 can correctly predict the 5-years survival rate of patients with CC, thus aiding individualized decision-making for patients with CC. Moreover, the results of this study also provide some direction for future fundamental research. However, the biological function and molecular mechanism of PLEKHA8P1 need further study.

## Data Availability Statement

The datasets generated for this study can be found at https://portal.gdc.cancer.gov.

## Author Contributions

CH designed the study, analyzed the data, and wrote the manuscript with contributions from all authors. CH and JZ collected data. ZZ provided critical comments for this paper. All authors read and approved the final version of the paper.

## Conflict of Interest

The authors declare that the research was conducted in the absence of any commercial or financial relationships that could be construed as a potential conflict of interest.
